# The Mütter Lectures on Surgical Pathology

**Published:** 1892-06

**Authors:** 


THE MUTTER LECTURES ON SURGICAL PATHOLOGY. Delivered before the College of Physicians of Philadelphia, 1890-91, by ROSWELL PARK, A. M., M. D., Professor of Surgery, Medical Department, University of Buffalo; Surgeon to the Buffalo General Hospital; Fellow of the German Congress of Surgeons, etc. Reprinted from the *Annals of Surgery*, Volumes XIII., XIV., and XV. Pp. viii.—293. St. Louis: J. H. Chambers & Co. 1892.

To Professor Park's many friends in Buffalo and elsewhere, who have had occasion to listen to but fragments of his course of lectures on Surgical Pathology, it will be welcome news to know that they have been republished in book form. The synopsis of these lectures have been published in the columns of this journal, and, from time to time, excerpts from some of the lectures, so that our readers are not altogether unfamiliar with the scope and general bearing of the various subjects treated. The excellent work done by the author in surgical pathology and bacteriology enables him not only to re-investigate and confirm the work done by others, but also to add light and knowledge on subjects heretofore illy understood. The chapters on Wound Infection and Suppuration, The Results of the Absorption of the Products of Wound Infection, and those on Mixed and Secondary Infection, deserve special mention for their originality and completeness. The chapter on Tetany and Tetanus is interesting from a neuro-surgical point of view, and is replete with the latest information on these subjects. On the whole, Prof. Park's work is a worthy companion to the many treatises which have lately appeared on surgical pathology and bacteriology. W. C. K.

